# Development of Scalable Elastic Gelatin Hydrogel Films Crosslinked with Waterborne Polyurethane for Enhanced Mechanical Properties and Strain Recovery

**DOI:** 10.3390/gels11010049

**Published:** 2025-01-08

**Authors:** Soon Mo Choi, Eun Joo Shin, Sun Mi Zo, Madhusudana Rao Kummara, Chul Min Kim, Anuj Kumar, Han Jo Bae, Ankur Sood, Sung Soo Han

**Affiliations:** 1 Research Institute of Cell Culture, Yeungnam University, Gyeongsan 38541, Republic of Korea; smchoi@ynu.ac.kr (S.M.C.); sunmizo@ynu.ac.kr (S.M.Z.); msraochem@yu.ac.kr (M.R.K.); anuj.budhera@gmail.com (A.K.); 2 Department of Chemical Engineering, Dong-A University, Busan 49315, Republic of Korea; sejoo6313@dau.ac.kr; 3 School of Chemical Engineering, Yeungnam University, Gyeongsan 38541, Republic of Korea; 4 School of Mechatronics Engineering, Gyeongsang National University, Jinju 52725, Republic of Korea; cm@gnu.ac.kr; 5 Department of Smart Fashion Material, Yeongnam Convergence Technology Campus of Korea Polytechnic, Daegu 41207, Republic of Korea; hjbae@kopo.ac.kr

**Keywords:** gelatin, waterborne polyurethane, crosslinking, hydrogel, urea, urethane

## Abstract

Exploiting novel crosslinking chemistry, this study pioneers the use of waterborne polyurethane (WPU) to chemically crosslink porcine-derived gelatin, producing enhanced gelatin hydrogel films through a solvent-casting method. Our innovative approach harnesses the reactive isocyanate groups of WPU, coupling them effectively with gelatin’s hydroxyl and primary amino groups to form robust urea and urethane linkages within the hydrogel matrix. This method not only preserves the intrinsic elasticity of polyurethane but also significantly augments the films’ tensile strength and strain. Comprehensive characterizations of these hydrogel films and pre-formed hydrogel reaction mixtures were conducted using viscosity measurements, Fourier Transform Infrared Spectroscopy (FTIR), Thermogravimetric Analysis (TGA), and the universal testing machine (UTM) for tensile-recovery assessments, alongside evaluations of their biocompatibility. The results demonstrated a reduction in pore size with an increase in WPU concentration from 2 to 6% in the developed hydrogels with a decrease in the equilibrium swelling ratio from 15% to 9%, respectively. Further, hydrogels with 6% WPU exhibited the highest tensile stress in both a dry and wet state. The gelatin hydrogel formed with 6% WPU blend also demonstrated the growth and proliferation of CCD-986K (fibroblast) and CCD-1102 (keratinocyte) cells for up to 5 days of co-culturing. The results indicate a notable enhancement in the mechanical properties and biocompatibility of gelatin hydrogels upon the introduction of WPU, positioning these films as superior candidates for biomedical applications such as tissue engineering and wound dressing.

## 1. Introduction

In the era of technological advancements, there is a constant inflow of information that leads to the development of noble materials with remarkable applications. Researchers across the globe have been working in the development of extremely efficient and high-performing biomaterials to eliminate dependencies on non-renewable polymeric materials [1,2]. Extensive research is underway to functionalize the biomaterials to provide cytocompatibility to these biomaterials [3]. Innate cells are surrounded by an extracellular matrix (ECM), which operates as a three-dimensional (3D) architect that regulates cell growth, function, differentiation, and maturation [4,5]. Considerable efforts are underway to mimic this innate 3D environment. There have been many biomaterial-based constructs that are of valuable importance in the field of biomedicine but out of all of them, hydrogels have been widely explored [6,7,8].

Hydrogels, known for their structural similarity to the extracellular matrix (ECM) and exceptional biocompatibility, have been a central focus in biomedical research [9,10]. Despite their potential, conventional gelatin-based hydrogels face inherent limitations, including poor mechanical strength, rapid swelling, and low elasticity, which hinder their widespread application [11,12]. Additionally, traditional crosslinking strategies often rely on toxic chemicals like glutaraldehyde or solvent-based methods, posing environmental and safety challenges [13,14]. To confront this challenge and address this concern, waterborne polyurethane (WPU) is considered as an ideal candidate that could be explored as an innovative and eco-friendly crosslinking agent to address these issues. The choice of WPU is supported by the fact that unlike the conventional polyurethanes, WPU has low volatile organic compounds, making it more environmentally friendly [15,16]. Unlike conventional crosslinkers, WPU operates within a solvent-free, non-toxic system, leveraging its reactive isocyanate (-NCO) groups to form robust urea (NH-CO-NH) and urethane (O-CO-NH) linkages [17,18]. This novel crosslinking chemistry not only eliminates the use of harmful chemicals but also enhances the mechanical robustness and elasticity of the resulting hydrogels. A key aspect in utilizing WPU is its ability to promote secondary hydrogen bonding alongside covalent interactions with the internal architect of the hydrogel networks [19]. These dual mechanisms enable the development of dense, durable hydrogel networks with tunable properties. By mitigating the limitations of conventional hydrogels, the proposed strategy significantly enhances mechanical strength, strain recovery, and thermal stability. This positions the hydrogels for use in advanced applications, such as tissue engineering and wound healing, where both mechanical integrity and biocompatibility are critical.

This study pioneers the use of waterborne polyurethane (WPU) as a dual crosslinker that can enhance the mechanical attributes and thermal performance of conventional gelatin-based hydrogels. The selection of gelatin for hydrogel formation was conducted owing to their unique physical, chemical, and biological features alongside their strong mechanical behavior and biocompatible nature [20,21]. Different blends of WPU and gelatin were fabricated to study the effect of WPU concentration on the development of a hydrogel network (GWPU1-3). Furthermore, the study adopts a simplified solvent-casting method for hydrogel fabrication, showcasing its industrial scalability and cost-effectiveness. The WPU-based gelatin hydrogels are physicochemically evaluated for their morphology and structural assessment. Furthermore, the biological response of the as-developed gelatin-based hydrogels was tested by co-culturing CCD-986K (fibroblast) and CCD-1102 (keratinocyte) cells and observing their adhesion, proliferation, and morphology over a 5-day period.

This streamlined approach addresses the complexity of existing manufacturing processes, offering practical solutions for large-scale production. By advancing a sustainable and biocompatible crosslinking strategy, this research bridges critical gaps in hydrogel design. It introduces a novel approach that not only meets the stringent requirements of biomedical applications but also aligns with environmental sustainability goals, setting a new benchmark for future hydrogel development.

## 2. Results and Discussion

### 2.1. Characterization of Gelatin-Based Hydrogel

The formation of gelatin hydrogels crosslinked with waterborne polyurethane (WPU) was thoroughly characterized through a combination of chemical and physical analyses.

**Scheme 1:**
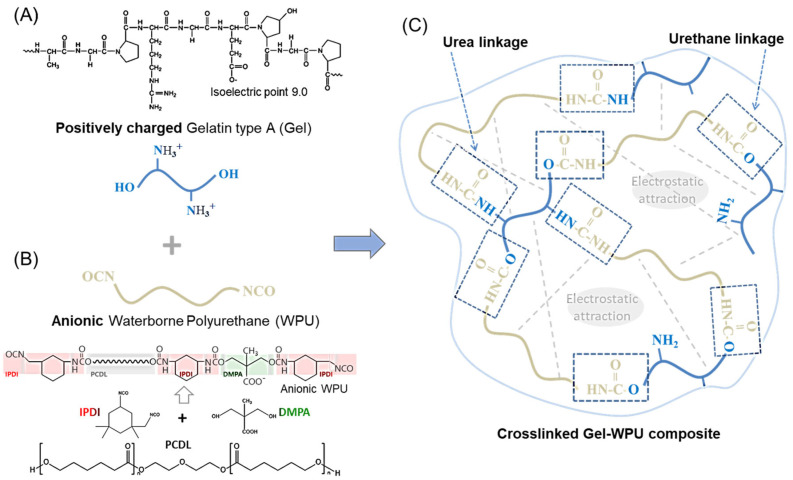
Reaction mechanism for gelatin hydrogel formation crosslinked by waterborne polyurethane (WPU). (**A**) Positively charged gelatin type A (Gel) structure, showing its cationic amino groups; (**B**) schematic representation of the anionic waterborne polyurethane (WPU) synthesis process, including the key components: isophorone diisocyanate (IPDI), dimethylolpropionic acid (DMPA), and polycarbonate diol (PCDL); (**C**) formation of the gelatin hydrogel crosslinked by WPU through the reaction between positively charged gelatin and anionic WPU. The electrostatic attractions and covalent bonding (via urea and urethane linkages) between amino groups (NH_2_) in gelatin and isocyanate groups (NCO) in WPU result in a robust, crosslinked hydrogel structure.

The reaction mechanism illustrated in Scheme 1 is governed by a synergistic combination of electrostatic interactions and chemical crosslinking, leveraging the distinct properties of gelatin and WPU. Gelatin, derived from porcine skin (type A), possesses an isoelectric point of approximately 9, rendering it positively charged at physiological pH (7.4) due to the protonation of amine groups (-NH_3_^+^) [22]. In contrast, WPU features negatively charged ionic groups, which engage in electrostatic interactions with the positively charged gelatin. These interactions facilitate the efficient mixing of the components and establish an initial network, stabilizing the hydrogel matrix. The chemical crosslinking process is primarily driven by the reactive isocyanate groups (-NCO) of WPU, which undergo nucleophilic addition reactions with the functional groups of gelatins. Specifically, it is speculated that the amine groups (-NH_2_) of gelatin form urea linkages (-NH-CO-NH), and the hydroxyl groups (-OH) of gelatin react to create urethane linkages (-O-CO-NH). These covalent bonds significantly enhance the structural integrity and mechanical robustness of the hydrogel. Notably, no residual isocyanate groups were detected in the final hydrogel, as confirmed by the absence of the characteristic -NCO peak in the FTIR spectra, indicating the completion of the reaction. Beyond covalent bonding, WPU also promotes extensive hydrogen bonding within the hydrogel matrix. It is speculated that these secondary interactions occur between the urea and urethane linkages, and between the residual functional groups on gelatin and WPU chains. The synergistic combination of covalent and secondary interactions enhances the hydrogel’s mechanical strength, elasticity, and overall stability, making it a promising candidate for biomedical applications.

**Figure 1:**
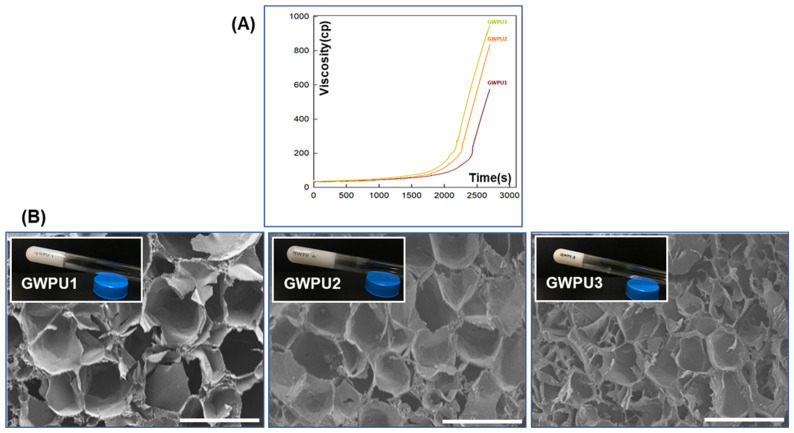
(**A**) Time-dependent viscosity curves of the gelatin-WPU blending solutions for GWPU1, GWPU2, and GWPU3, (**B**) SEM micrographs showing the internal microstructure of the fabricated hydrogels GWPU1, GWPU2, and GWPU3, respectively. The inset in each SEM micrograph represents a digital image of the corresponding hydrogel samples (scale bar: 500 µm).

In order to make hydrogels, the crosslinking and the viscoelastic properties of the blending solution are very crucial in establishing the capability of the blending solution to develop a mechanically robust hydrogel network [23,24]. Changes in the viscosity of the gelatin and WPU blending solutions could provide critical insights into the composition and interactions prior to hydrogel formation. Figure 1A illustrates the time-dependent viscosity changes of the samples labeled as GWPU1, GWPU2, and GWPU3. Initially, all three samples exhibit a similar trend in the time-dependent viscosity behavior; however, a significant increase in the viscosity was observed over the passage of time. This increment in the viscosity supports the predicted chemical interactions and crosslinking between the isocyanate (-NCO) groups of WPU and the amino (-NH_2_) and hydroxyl (-OH) groups of gelatins. Further, this increase in the viscosity reflects a WPU concentration-dependent behavior which in turn is governed by the extent of crosslinking reactions. Specifically, GWPU1 shows the slowest viscosity rise, suggesting limited crosslinking due to its lower WPU concentration. In contrast, GWPU3 exhibits the steepest viscosity increase, indicating that higher WPU content promotes more extensive crosslinking reactions, resulting in a pronounced rise in viscosity. This increase corroborates the formation of strong chemical bonds between WPU’s isocyanate groups and gelatin, thereby enhancing the network structure of the mixture. It is also speculated that there could be a physical interaction (electrostatic interaction, hydrogen bonding) between the gelatin solution and WPU solution, leading to increased viscosity in GWPU1-3. These results confirm the uniformity of the initial blending solution and its reactivity, providing critical information on the extent of crosslinking for gelation and WPU-dependent viscosity behavior. These parameters are very critical for optimizing the composition of the blending solution to achieve hydrogel with tailored mechanical properties and structural stability. The viscosity analysis thus offers valuable data for customizing the gelatin–WPU mixture to develop hydrogels with superior performance.

Further, in order to analyze the pore architectural behavior for the developed hydrogel networks, SEM was employed. Figure 1B presents SEM images showcasing the internal microstructures of gelatin hydrogels crosslinked using WPU as a crosslinker. The samples, labeled GWPU1, GWPU2, and GWPU3, were synthesized with varying WPU concentrations, and the SEM images provide critical insights into the effects of WPU concentration on the microstructural properties of the hydrogels. GWPU1 prepared with the lowest WPU concentration (2 wt.%) exhibits relatively large pores. The loose and open network structure reflects the lower crosslinking density, resulting in increased free space within the hydrogel matrix [25]. This structural characteristic suggests that the initial crosslinking reactions in the blending solution were limited due to the reduced availability of WPU, leading to a less compact hydrogel network. GWPU2 synthesized with a WPU 4 wt.% shows a noticeable reduction in pore size and an increase in structural density compared to GWPU1. The higher WPU content enhances crosslinking activity, producing a more interconnected and denser network. This structural transformation likely contributes to the improved mechanical strength and stability of the hydrogel. GWPU3 fabricated with the highest WPU 6 wt.% demonstrates the smallest pores and the highest network density. The increased WPU availability facilitates extensive crosslinking, maximizing the structural integrity of the hydrogel. This enhanced crosslinking results in a more stable and mechanically robust network, underscoring the significant role of WPU concentration in determining the hydrogel’s properties. These SEM images visually confirm the progressive changes in pore size and network density across the samples, highlighting the structural impact of varying WPU concentrations. As the WPU content increases, the crosslinking density rises, leading to a tighter network with smaller pores. This trend reflects the effectiveness of the crosslinking process in tailoring the hydrogel’s microstructure and its subsequent mechanical properties. It is to be noted that the appearance of the hydrogel samples (presented as inset in the SEM images) does not present any significant difference, as all three variants were able to form hydrogels. Overall, SEM analysis provides a comprehensive understanding of the structural evolution in gelatin hydrogels crosslinked with WPU. The ability to manipulate pore size and network density through WPU concentration underscores the versatility of this crosslinking strategy, paving the way for designing hydrogels with optimized mechanical stability and tailored properties for diverse applications.

**Figure 2:**
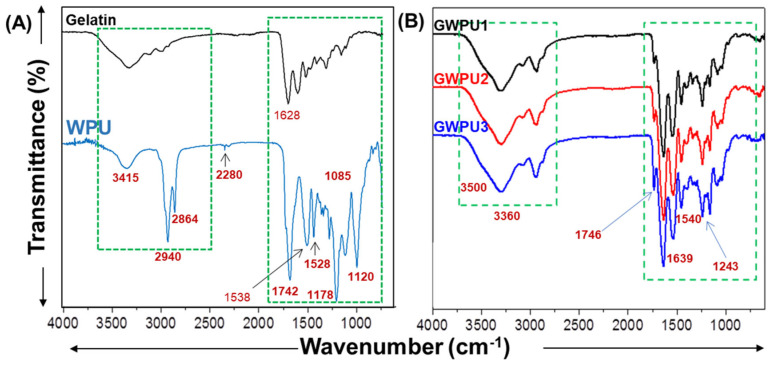
FTIR spectra of gelatin, WPU, and gelatin hydrogel crosslinked by WPU composites (GWPU1, GWPU2, and GWPU3). FTIR spectra of (**A**) waterborne polyurethane (WPU) and gelatin, (**B**) the gelatin hydrogel crosslinked by WPU (GWPU1, GWPU2, and GWPU3).

To further analyze the bonding pattern of the developed hydrogels, FTIR analysis was conducted. Figure 2A shows the FTIR spectra of the pure components and the possible chemical interactions between the components in the composite systems. Pure gelatin showed main characteristic peaks at around 3291 cm^−1^ (N–H coupling with –OH groups), 1626 cm^−1^ assigned to C=O/C–N stretching (amide I) and hydrogen bonding coupled with COO– stretching for GE, and 1529 cm^−1^ (N–H bending, amide II) [26,27]. Further, WPU showed characteristic peaks at around 3415 cm^−1^ (N–H stretching), symmetric and asymmetric stretching peaks for aliphatic C-H stretching were observed at around 2940 cm^−1^ and 2864 cm^−1^, respectively (the isocyanate), the terminal group appeared as a very minute peak at around 2280 cm^−1^, which could be attributed to the out of phase –N=C=O stretch, 1742 cm^−1^ (urethane C=O stretching), 1538 cm^−1^ (urethane N-H bending), and 1000–1180 cm^−1^ (C–O–C stretching) [28,29]. In the case of GWPU samples (Figure 2B), the broad peak at around 3300–3400 cm^−1^ was observed due to the intramolecular and intermolecular H-bonding between gelatin and WPU molecules and chains [29,30]. The characteristic peaks for –NH stretching (urethane and gelatin) for GWPU1, GWPU2, and GWPU3 were observed at around 3356 cm^−1^, 3341 cm^−1^, and 3338 cm^−1^, respectively, while those of the C=O groups (urethane, –NHCOO–) were around 1746 cm^−1^ for all GWPU samples. In addition, the characteristic peak at around 1746 cm^−1^ for –NHCOO-gelatin also appeared in GWPU1, GWPU2, and GWPU3. The characteristic peak of the C=O groups (1727 cm^−1^) from the urethane bonds in pure WPU shifted to 1746 cm^−1^ in the GWPU composites due to –NHCOO-gelatin linkage. As compared to pre-polymers, GWPU1 exhibited an increase in the intensity of the peak at around 3356 cm^−1^ for N–H stretching and one new peak appeared at around 1639 cm^−1^ (C=O stretching, amide I) and hydrogen-bonded urea (C=O), while increasing with the increased gelatin content in the pre-polymer systems (GWPU2 and GWPU3). In addition, it also may be due to H-bonded urea C=O groups [31,32]. The changes support the formation of new urea/urethane bonds (NH–C=O–NH (urea linkage) and O–C=O–NH (urethane linkage) [33] due to the reaction of gelatin and WPU in the GWPU systems [28]. In addition, no peak at around the 2265 cm^−1^ peak was observed, revealing that GWPU composites do not possess free –NCO groups of the WPU precursors [34]. The absence of the characteristic isocyanate peak in the GWPU composites confirms the complete reaction between WPU and gelatin. This demonstrates that WPU’s reactive isocyanate groups efficiently form covalent bonds with gelatin’s functional groups, eliminating any residual –NCO groups and ensuring the safety and stability of the final hydrogel. It is a critical advantage for biomedical applications. Further, the peaks at 1540 cm^−1^ (C=O stretching, amide II and hydrogen-bonded urea (C=O)) and 1243 cm^−1^, (amide III) of gelatin overlap with the urethane and urea N-H deformation and C–N and C–O stretching in the GWPU composites, respectively [28]. These peaks could not provide any critical distinction to further support the composite formation in GWPU samples.

**Figure 3:**
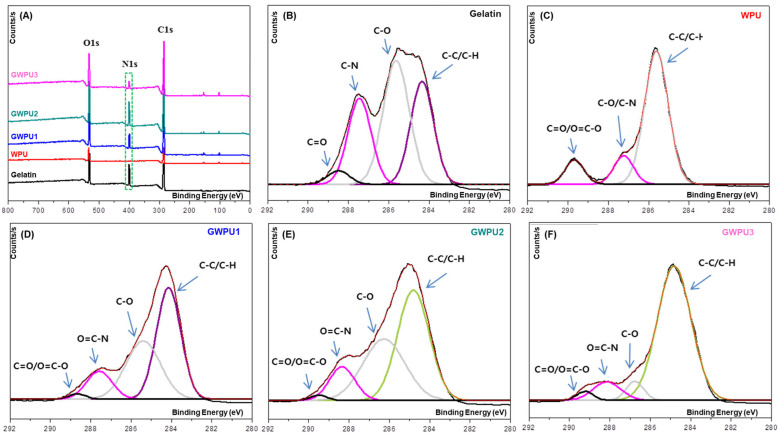
XPS analysis of gelatin, WPU, and gelatin hydrogel crosslinked by WPU composites (GWPU1, GWPU2, and GWPU3). (**A**) Survey spectra showing the elemental composition of gelatin, WPU, and gelatin hydrogel crosslinked by WPU composites (GWPU1, GWPU2, and GWPU3), and the fitting curves of XPS C 1s spectra are presented for (**B**) gelatin, highlighting carbonyl (C=O) and hydroxyl (C–O) groups, (**C**) WPU, indicating urethane (C=O) and aliphatic carbon (C–C) groups, and (**D**–**F**) GWPU1, GWPU2, and GWPU3, showing the progression of urea and urethane bond formation.

The XPS analysis was conducted to evaluate the chemical interactions between the gelatin and WPU in the composites. Complementing the FTIR results, XPS provides direct evidence of the chemical bonding between gelatin and WPU. Figure 3 presents the XPS survey scans and high-resolution C1s spectra of pure gelatin, WPU, and their composites (GWPU1, GWPU2, and GWPU3). The survey scans (Figure 3A) reveal the presence of characteristic peaks for carbon (C1s), nitrogen (N1s), and oxygen (O1s) at approximately 285 eV, 400 eV, and 531 eV, respectively, indicating that these three elements are common across all samples. The high-resolution XPS C1s spectra of pure gelatin (Figure 3B) showed four distinct peaks at 284.7 eV (C=C/C–C), 285.6 eV (C-OH), 286.3 eV (C–N), and 288.6 eV (C=O), which is in coherence with the reported results for the deconvolution of C1s in the case of gelatin [27]. In contrast, the high-resolution scan of C1s of pure WPU (Figure 3C) exhibited three peaks at 285.6 eV (C=C/C–C), 287.3 eV (C–O/C–N), and 289.8 eV (C=O/O=C–O), indicative of its urethane structure [35,36]. For GWPU1 (Figure 3D), the C1s spectra displayed four peaks at 284.2 eV (C=C/C–C), 285.4 eV (C–O), 287.6 eV (O=C–N), and 288.7 eV (C=O/O=C–O), confirming the coexistence of gelatin and WPU components and the formation of new urea (NH–C=O–NH) and urethane (O–C=O–NH) linkages in the composite film [33]. Notably, in GWPU1, the oxygen (O1s) peak intensity increased, while the nitrogen (N1s) peak intensity decreased compared to pure gelatin or WPU, which could be attributed to the enhanced interactions between the components [37]. In the case of GWPU2, the C1s high-resolution spectrum showed peaks at 284.8 eV (C=C/C–C), 286.4 eV (C–O), 288.4 eV (O=C–N), and 289.5 eV (C=O/O=C–O), whereas GWPU3 exhibited peaks at 284.9 eV (C=C/C–C), 286.8 eV (C–O), 288.2 eV (O=C–N), and 289.3 eV (C=O/O=C–O). These shifts and intensities indicate increasing chemical interactions with higher gelatin content in the composite films. Overall, the XPS analysis confirms the formation of covalent bonds (urea and urethane linkages) between GE and WPU, alongside increased physical crosslinking (hydrogen bonding) in GWPU3 compared to GWPU1 and GWPU2. This network imparts excellent mechanical properties, strain recovery, and elasticity to the hydrogel, which are further discussed in the section on tensile testing and swelling behavior.

### 2.2. Mechanical Characterization of Gelatin-Based Hydrogel

**Figure 4:**
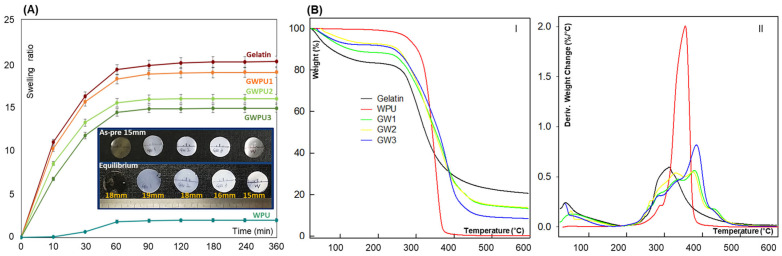
(**A**) Swelling behavior of gelatin, WPU, and gelatin hydrogel crosslinked by WPU composites (GWPU1, GWPU2, and GWPU3). The inset shows the dimensional changes of the composites before swelling (as-prepared, 15 mm diameter) and after reaching equilibrium swelling. (**B**) Thermal stability analysis using thermogravimetric analysis (TGA). (**I**) TGA weight loss curves and (**II**) Derivative weight loss (DTG) of gelatin, WPU, and gelatin hydrogel crosslinked by WPU composites (GWPU1, GWPU2, and GWPU3).

The degree of swelling is one of the most important parameters that governs the release pattern from the hydrogel network. Figure 4A illustrates the swelling behavior of gelatin, WPU, and GWPU composites (GWPU1, GWPU2, and GWPU3) over time, providing insight into the impact of WPU content on hydrogel network structure and water uptake. The swelling ratio of gelatin shows a significant increase within the initial 30 min, reaching equilibrium at an approximate swelling ratio of 20. This behavior could be attributed to its highly hydrophilic nature and lack of crosslinking [38]. In the case of WPU, the swelling ratio is very low, which could be attributed to the high crosslink density of WPU, which restricts its ability to absorb water [39]. In contrast, the GWPU hydrogels exhibit a lower swelling ratio compared to pure gelatin, which decreases as the WPU content increases. GWPU1, GWPU2, and GWPU3 achieve equilibrium swelling ratios of approximately 15, 12, and 9, respectively, reflecting the densification of the hydrogel network with higher WPU crosslinking density. It is further speculated that the reduced swelling is attributed to the covalent crosslinking provided by WPU, which limits water absorption and stabilizes the hydrogel structure. Meanwhile, the WPU-only sample exhibits the lowest swelling ratio due to its dense and rigid network structure. These results confirm that increasing WPU content effectively modulates the swelling behavior of the hydrogels, enhancing their structural integrity and reducing excessive water uptake, which is critical for applications requiring mechanical durability and controlled hydration. In order to test the physical and chemical features of all native and composite samples as a function of temperature, thermogravimetric analysis (TGA) was used. Figure 4B illustrates the TGA results of the gelatin, WPU, and GWPU composites (GWPU1, GWPU2, and GWPU3). The weight loss profiles reveal three distinct degradation stages across all samples. The first stage, occurring at around 130 °C, is attributed to the evaporation of residual moisture and bound water. The second stage, between 200 °C and 350 °C, corresponds to the decomposition of organic components such as gelatin and the urethane/urea linkages within the hydrogels [40]. The final stage, above 400 °C, represents the complete thermal degradation of the polymer backbone [41]. Notably, GWPU hydrogels exhibit a thermal degradation pattern in trend with pure gelatin. On comparison, the total residual remains for GWPU 1, GPWU2, and GWPU3 are approximately 17.9%, 17.5%, and 16.4%, respectively. Among the GWPU samples, GWPU3 demonstrates the highest thermal stability, reflecting the denser network structure formed due to its higher WPU content. These findings underscore the role of WPU as an effective crosslinker, improving both the mechanical and thermal properties of the hydrogel system.

**Figure 5:**
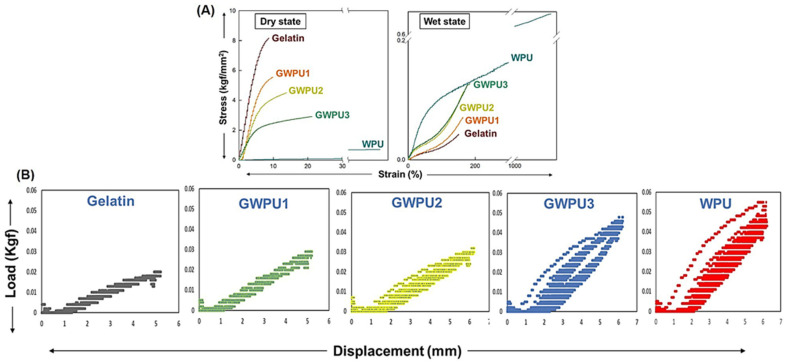
(**A**) Stress–strain curves of gelatin, WPU, and GWPU composites in both dry and wet states, highlighting the mechanical properties and strain recovery. (**B**) Load–displacement curves representing the elastic deformation behavior of gelatin, WPU, and GWPU composites in wet state.

The tensile strength and recovery properties of the gelatin, WPU, and GWPU composites were evaluated through stress–strain and load–displacement analyses, as shown in Figure 5. Figure 5A shows the stress–strain curves depicting the mechanical behavior of the materials under both dry and wet conditions. In the dry state, pure gelatin exhibited the highest tensile stress but the lowest elongation at break, indicating its brittle nature and limited flexibility. In contrast, WPU displayed the highest elongation at break but substantially lower tensile stress, reflecting its inherent flexibility and elasticity. The GWPU composites demonstrated a synergistic enhancement in mechanical properties, effectively combining the stiffness of gelatin with the elasticity of WPU. Among the GWPU samples, GWPU3 exhibited the highest tensile stress, underscoring the influence of increased WPU concentration on crosslinking density and structural reinforcement. In the wet state, all samples showed a notable reduction in tensile stress due to water absorption, which disrupted intermolecular interactions. However, GWPU composites, particularly GWPU3, maintained superior mechanical integrity compared to gelatin, attributed to the robust network of urea and urethane linkages formed during crosslinking. These findings indicate that incorporating WPU not only enhances mechanical strength but also mitigates performance loss under hydrated conditions. Figure 5B presents the load–displacement curves under wet conditions, focusing on the elastic deformation behavior of the samples. All samples exhibited a progressive increase in load capacity with displacement. WPU showed the highest load tolerance, highlighting its excellent elasticity. Among the composites, GWPU3 demonstrated the best elastic recovery, attributed to its enhanced crosslinking density and the balance between rigidity and flexibility provided by WPU. The gradual load increase observed in GWPU composites further validates their superior resistance to deformation under applied stress. These results confirm that GWPU composites exhibit a well-balanced mechanical performance, with enhanced tensile strength, flexibility, and strain recovery. The ability to tune these properties by varying the WPU concentration positions GWPU composites as a promising candidate for biomedical applications, including wound dressings and tissue engineering scaffolds.

### 2.3. Cell Viability and Proliferation on Prepared Gelatin-Based Hydrogels

**Figure 6:**
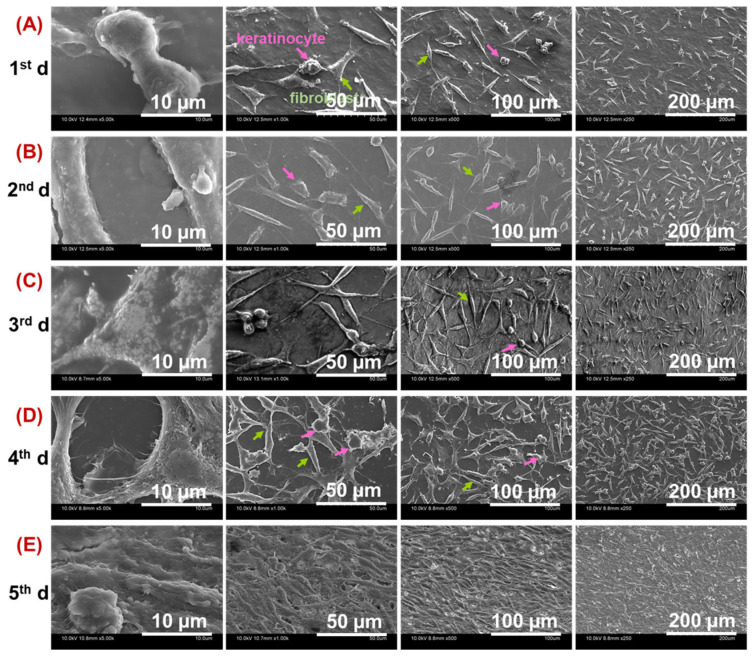
SEM images depicting the adhesion, proliferation, and morphology of CCD-986K (skin fibroblast) and CCD-1102 (keratinocyte) cultured on GWPU composite over 5-day period. (**A**) Day 1: initial adhesion of fibroblasts (green arrow) and keratinocytes (pink arrows). (**B**) Day 2: increased cell spreading and network formation. (**C**) Day 3: enhanced proliferation with distinct morphological features of fibroblasts and keratinocytes. (**D**) Day 4: advanced cellular interaction and dense cellular coverage. (**E**) Day 5: fully developed cellular network indicating successful proliferation and attachment. Fibroblasts and keratinocytes are differentiated based on their characteristic morphologies, highlighted by green and pink arrows, respectively.

The physicochemical, thermal, and mechanical evaluation of GWPU composites presented the high superiority of GWPU3 hydrogels, which was further selected to test their biological response. The biocompatibility of the GWPU3 composite was evaluated by co-culturing CCD-986K (fibroblast) and CCD-1102 (keratinocyte) cells and observing their adhesion, proliferation, and morphology over a 5-day period, as illustrated in Figure 6. The use of SEM enabled the distinct identification of fibroblasts and keratinocytes based on their characteristic morphologies, despite being co-cultured on the same hydrogel surface. Fibroblasts exhibited elongated, spindle-like shapes with extended filopodia (green arrows), whereas keratinocytes displayed more polygonal, flattened morphologies (pink arrows) [42]. Fibroblasts and keratinocytes successfully attached to the GWPU3 hydrogel on Day 1, initiating small, scattered clusters. Over the subsequent days, fibroblasts formed interconnected networks while keratinocytes spread into larger patches, maintaining distinct morphologies. By Day 4, the cells extensively covered the hydrogel, with fibroblasts creating a supportive matrix and keratinocytes forming a confluent monolayer. On Day 5, the two cell types established a fully cohesive structure, mimicking the natural dermis– epidermis interaction and demonstrating the biocompatibility and suitability of GWPU3 for co-culture systems and tissue engineering applications.

**Figure 7:**
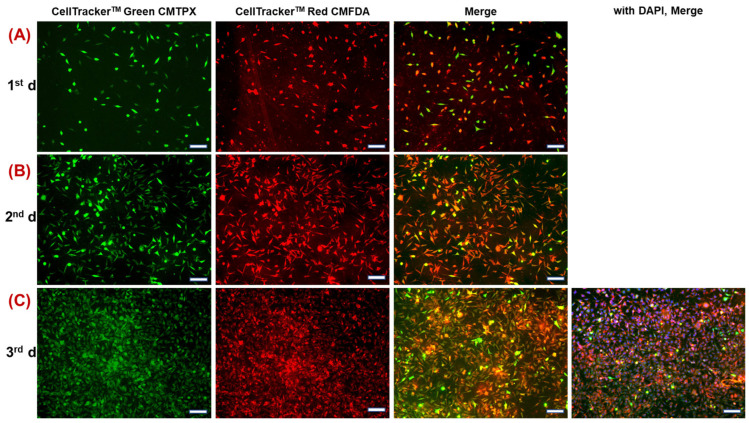
Fluorescent images illustrating the adhesion, proliferation, and morphology of CCD-986K (skin fibroblast) and CCD-1102 (keratinocyte) cultured on GWPU composite over 3-day period. (**A**) Day 1: initial cell adhesion observed with CellTracker™ Green CMTPX for fibroblasts and CellTracker™ Red CMFDA for keratinocytes, with merged images confirming co-culture. (**B**) Day 2: increased cell proliferation and spreading, showing enhanced signal intensity and distinct cellular interactions in merged images. (**C**) Day 3: dense and well-distributed cellular growth, with overlay images (including DAPI staining) indicating successful cell proliferation and nuclei staining. Each color corresponds to specific cell types: green for fibroblasts, red for keratinocytes, and blue for nuclei. (Scale bar: 100 µm).

Fluorescent labeling using CellTracker™ Red CMTPX (fibroblasts), CellTracker™ Green CMFDA (keratinocytes), and DAPI (nuclei) was employed to visualize the spatial distribution and proliferation behavior of the cells on the GWPU3 composite surface. The fluorescence images presented in Figure 7 represent the experimental results from days 1, 2, and 3 of co-culture. This time frame corresponds to the effective working period of the CellTracker™ days, which function optimally for up to 72 h after staining the cells. The observed results illustrate the progression of cell attachment, proliferation, and interaction on the GWPU3 hydrogel scaffold over these three days. On the 1st day, fibroblasts (red) and keratinocytes (green) successfully adhered to the GWPU3 hydrogel surface, displaying distinct morphologies. After that, both cell types demonstrated significant proliferation, forming dense clusters and interconnected networks. The overlap of red and green fluorescence (yellow regions) in merged images highlights close interactions between the two cell types. This indicates that GWPU3 provides a suitable environment for the simultaneous growth and spatial arrangement of fibroblasts and keratinocytes. Fibroblasts established a structural framework across the hydrogel, while keratinocytes formed a continuous layer on top, resembling the dermis–epidermis architecture of natural skin. The DAPI staining confirmed a uniform nuclear distribution, emphasizing the viability and stable growth of cells on the hydrogel.

## 3. Conclusions

This study presents a pioneering approach to developing gelatin-based hydrogels chemically crosslinked with waterborne polyurethane (WPU) without additional crosslinking agents. The successful integration of gelatin and WPU was achieved through synergistic electrostatic and covalent interactions, forming robust networks characterized by urea and urethane linkages. This innovative strategy preserves the intrinsic elasticity of WPU while significantly enhancing the hydrogel’s mechanical properties, including tensile strength and strain recovery. Comprehensive analyses, including FTIR, XPS, and SEM, confirmed the complete reaction between gelatin and WPU, with no residual isocyanate groups, ensuring the safety and stability of the hydrogels for biomedical applications.

The tunable microstructure, as observed through SEM, allowed control over pore size and crosslinking density, demonstrating the flexibility of this system to tailor hydrogels for specific needs. Furthermore, the superior thermal stability and reduced swelling ratios highlight the improved durability and structural integrity of the WPU-crosslinked hydrogels. Biocompatibility assessments using CCD-986K fibroblasts and CCD-1102 keratinocytes provided compelling evidence of the hydrogel’s suitability for tissue engineering applications. The co-culture experiments revealed the ability of the GWPU3 hydrogel to support cell adhesion, proliferation, and spatial organization, mimicking the dermis–epidermis structure of natural skin. The progression of cell networks over five days further underscores the material’s potential as a scaffold for skin regeneration.

In conclusion, this study introduces a sustainable, highly versatile, and biocompatible gelatin-based hydrogel system that meets the demands of biomedical applications such as wound dressings and tissue engineering scaffolds. Furthermore, the scalability and simplicity of the developed manufacturing process highlight its strong potential for industrial applications, particularly in large-scale hydrogel production. The innovative use of waterborne polyurethane as a crosslinking agent represents a significant advancement in hydrogel technology, offering new possibilities for environmentally friendly and high-performance biomaterials.

## 4. Materials and Methods

### 4.1. Materials

Gelatin type A from porcine skin, and 3-(4,5-dimethylthiazol-2-yl)-2,5-diphenyl-2H tetrazolium bromide (MTT), Polycaprolactone diol (Mn~530), dimethylol propionic acid (DMPA), isophorone diisocyanate (IPDI), D9542-10MG DAPI (2-(4-Amidinophenyl)-6-indolecarbamidine dihydrochloride, and 4′,6-Diamidino-2-phenylindole dihydrochloride were purchased from Sigma-Aldrich Co. (St. Louis, MO, USA). Acetone (C_3_H_6_O), and triethylamine (TEA) were purchased from Duksan Pure Chemical Co., Ltd. (Seoul, Republic of Korea). C34552 CellTracker™ Red CMTPX Dye and C2925 CellTracker™ Green CMFDA (5-Chloromethylfluorescein Diacetate) were purchased from Invitrogen™, Thermofisher Scientific, Waltham, MA, USA). All chemicals were used as received. All the experiments were conducted with double distilled (DI) water.

### 4.2. Synthesis of Water Polyurethane Dispersion (WPU)

WPU was synthesized according to the reported protocol optimized in our lab [43]. Briefly, in a four-necked flask, polycaprolactone diol (24.0 g) and DMPA (1.6 g) were introduced and kept under reflux condition with constant stirring. The reaction mixture was heated till 80 °C, until the polycaprolactone diol was completely melted. IPDI (9.6 g) was then added dropwise, and the reaction was continued under inert conditions for 2 h. Subsequently, 100 mL of acetone was introduced to the reaction mixture to reduce the viscosity of the prepolymer, and the solution was cooled to 60 °C. In the next step, the carboxylic groups of DMPA was neutralized with TEA (1.2 g) for 30 min, and the final product was dispersed in DI water with vigorous stirring for overnight at RT. Post stirring, the suspension volume was reduced to a WPU solid content of 25 wt.% by rotary vacuum evaporation at 30 °C to remove the acetone.

### 4.3. Synthesis of Gelatin Hydrogels

First, 10 wt.% gelatin solution was prepared in 180 mL and then divided into three separate beakers carrying 60 mL each. WPU dispersion of varying concentrations (2 mL, 4 mL, and 6 mL) was added to the three beakers denoted as WPU1, WPU2, and WPU3, respectively. The reaction mixtures were thoroughly mixed and kept undisturbed at room temperature for overnight. The reaction mixtures were poured in 24-well plates followed by curing at 60 °C for 3 h, resulting in three batches of gelatin WPU-based hydrogels designated as GWPU1, GWPU2, and GWPU3, respectively. The fabricated hydrogels were washed (three times) with distilled water at room temperature to remove any unreacted materials.

### 4.4. Characterization

#### 4.4.1. Rheological Analysis

Rheological analysis of the hydrogel samples was performed by using a rheometer (Physica MCR301; Anton Paar, Graz, Austria) with stainless-steel parallel-plate geometry (PP25). An angular frequency sweep in the range from 0.1 1/s to 100 1/s was carried out at 25 °C and a fixed strain amplitude (λ = 1.0%) under a linear viscoelastic domain.

#### 4.4.2. SEM

The morphology and the microstructure of the hydrogels were observed by a scanning electron microscopy (SEM; HITACHI S4800, Hitachi Ltd., Tokyo, Japan). Prior to analysis, hydrogels were equilibrated with distilled water and freeze-dried at −80 °C. For analysis, the samples were mounted on the stub and then coated with a thin layer of platinum at a low deposition rate.

#### 4.4.3. FTIR

The porous films were examined for bonding patterns by FTIR -ATR mode (Spectrum 100; PerkinElmer, Waltham, MA, USA) in transmittance mode, with samples in its native form. The spectra were recorded at RT over the spectral range of 4000–600 cm^−1^, with a resolution of 4 cm^−1^.

#### 4.4.4. XPS

The X-ray photoelectron spectra (XPS) of the films were obtained using XPS system (Thermo Fisher Scientific, Waltham, MA, USA), with a monochromatic aluminum K-alpha X-ray source. The square shaped samples (10 × 10 mm^2^) were subjecting to etching for 30 s using argon gas before analysis. Survey spectra were acquired with a pass energy of 200 eV and 50 eV for high resolution spectra.

#### 4.4.5. Swelling

The relative swelling behavior (%) was calculated by completely submerging the hydrogel samples (in the dried form) in PBS under room temperature. After that, the completely swollen hydrogel samples were removed from the PBS and the excess PBS on the surface of the samples was wiped out with the help of lint-free tissue wipes and the corresponding weight was recorded. The relative swelling ratio (%) was determined by using the following formula:

$$\mathrm{Relative}\;\mathrm{swelling}\;\mathrm{ratio}\;(\%)=\frac{Ws-Wd}{Wd}\times 100$$

#### 4.4.6. TGA

TGA for the hydrogel samples was carried out using a TG analyzer (Q600 SDT: TA Instruments, Seoul, Republic of Korea) with a heating rate of 10 °C/min in the range of 30 to 800 °C under an N_2_ atmosphere.

#### 4.4.7. Mechanical Behavior

The tensile strength was measured by an autograph tester (Instron 4201, Sidmazu, Shizuoka-shi, Japan). The width and length of the samples were set to 5 mm and 20 mm, respectively, with a dumbbell shape, respectively, and the test speed was set to 100 mm/min.

#### 4.4.8. Recovery

The tensile loading and unloading cycles were performed using a universal testing machine (UTM; Lloyd Instruments, West Sussex, UK) for cylindrical samples (diameter 20 mm and height 9 mm) at a maximum strain of 50% with a constant speed of 130 mm/m.

#### 4.4.9. Cell Biocompatibility

Hydrogel samples were seeded with CCD-986K fibroblasts and CCD-1102 keratinocytes (co-cultured) for SEM analysis (cell adhesion and spreading), Live/Dead assay (cell viability), and MTT assay (cell proliferation).

For cell culture, CCD-986K fibroblasts and CCD-1102 keratinocytes were co-cultured in Dulbecco Minimum Essential Media (DMEM) supplemented with 10% Fetal Bovine Serum (FBS) and 1% antibiotic combination while maintained in an incubator at 37 °C and 5% CO_2_. Prior to the co-culturing of cells on the hydrogels, the samples were sterilized by saturating in ethanol (70%), followed by washing three times with PBS. Then, the hydrogel samples were placed in two separate 24-well plates (in triplicate) and growth media were added followed by culturing the cells with a cell density of (5 × 104 cell/well for each cell type). The 24-well plates were then placed in an incubator for cell growth and proliferation.

At a specific time period, the cells were fixed using 2% Gluteraldehyde (Sigma-Aldrich) with an incubation time of 20 min at 4 °C. Post fixation, the cells were washed using cold PBS (3 times) at room temperature and were subjected to gradient dehydration using 30%, 50%, 70%, 80%, 90%, and twice with 100% ethanol (Sigma-Aldrich) while maintaining an incubation of 3 min at each concentration. After that, the samples were transferred to carbon taped aluminum stubs of 12.7 mm × 8 mm (Ted Pella, Inc., Redding, CA, USA), air dried at room temperature for 15–20 min and then sputter-coated with a thin layer of gold for 60 s with a sputter current of 20 mA. Images were acquired using Hitachi S-4800 SEM at an operating voltage of 15 kV.

For qualitative analysis, the hydrogel samples in the 24-well plate were washed with PBS and stained with the following dyes: 1 µL/well of Red CMFDA (10 mmol), 1 µL/well of Green CMTPX (10 mmol), followed by incubation for 30 min, and 1 µL/well of DAPI (1 µg/mL) incubated for 10 min.

#### 4.4.10. Statistical Analysis

Three independent experiments were conducted and the final data representation includes the mean standard deviation (SD). The student t-test was conducted to calculate statistically significant values.

## Data Availability

The data presented in this study are openly available in the article.

## References

[1] Zhao S., Pang H., Li Z., Wang Z., Kang H., Zhang W., Zhang S., Li J., Li L. (2021). Polyurethane as high-functionality crosslinker for constructing thermally driven dual-crosslinking plant protein adhesion system with integrated strength and ductility. Chem. Eng. J..

[2] Forrester M., Becker A., Hohmann A., Hernandez N., Lin F.-Y., Bloome N., Johnson G., Dietrich H., Marcinko J., Williams R.C. (2020). RAFT thermoplastics from glycerol: A biopolymer for development of sustainable wood adhesives. Green Chem..

[3] Wang Y., Zhai W., Cheng S., Li J., Zhang H. (2023). Surface-functionalized design of blood-contacting biomaterials for preventing coagulation and promoting hemostasis. Friction.

[4] Sood A., Ji S.M., Kumar A., Han S.S. (2022). Enzyme-Triggered Crosslinked Hybrid Hydrogels for Bone Tissue Engineering. Materials.

[5] Asim S., Hayhurst E., Callaghan R., Rizwan M. (2024). Ultra-low content physio-chemically crosslinked gelatin hydrogel improves encapsulated 3D cell culture. Int. J. Biol. Macromol..

[6] Cao H., Duan L., Zhang Y., Cao J., Zhang K. (2021). Current hydrogel advances in physicochemical and biological response-driven biomedical application diversity. Signal Transduct. Target. Ther..

[7] Hoffman A.S. (2012). Hydrogels for biomedical applications. Adv. Drug Deliv. Rev..

[8] Zhou J., Zhang Y., Zhang M., Yang D., Huang W., Zheng A., Cao L. (2024). High-Performance MXene Hydrogel for Self-Propelled Marangoni Swimmers and Water-Enabled Electricity Generator. Adv. Sci..

[9] Choudhary A., Sharma A., Singh A., Han S.S., Sood A. (2024). Strategy and Advancement in Hybrid Hydrogel and Their Applications: Recent Progress and Trends. Adv. Eng. Mater..

[10] Singhmar R., Son Y., Jo Y.J., Zo S., Min B.K., Sood A., Han S.S. (2024). Fabrication of alginate composite hydrogel encapsulated retinoic acid and nano Se doped biphasic CaP to augment in situ mineralization and osteoimmunomodulation for bone regeneration. Int. J. Biol. Macromol..

[11] Lin X., Zhao X., Xu C., Wang L., Xia Y. (2022). Progress in the mechanical enhancement of hydrogels: Fabrication strategies and underlying mechanisms. J. Polym. Sci..

[12] Yuk H., Wu J., Zhao X. (2022). Hydrogel interfaces for merging humans and machines. Nat. Rev. Mater..

[13] Kojic N., Panzer M.J., Leisk G.G., Raja W.K., Kojic M., Kaplan D.L. (2012). Ion electrodiffusion governs silk electrogelation. Soft Matter.

[14] Maiti S., Maji B., Yadav H. (2024). Progress on green crosslinking of polysaccharide hydrogels for drug delivery and tissue engineering applications. Carbohydr. Polym..

[15] Shin E.J., Choi S.M. (2018). Advances in Waterborne Polyurethane-Based Biomaterials for Biomedical Applications. Adv. Exp. Med. Biol..

[16] Hsieh C.-T., Hsu S.-h. (2019). Double-Network Polyurethane-Gelatin Hydrogel with Tunable Modulus for High-Resolution 3D Bioprinting. ACS Appl. Mater. Interfaces.

[17] Rodrigues I.C.P., Woigt L.F., Pereira K.D., Luchessi A.D., Lopes É.S.N., Webster T.J., Gabriel L.P. (2020). Low-cost hybrid scaffolds based on polyurethane and gelatin. J. Mater. Res. Technol..

[18] Díez-García I., Lemma M.R.d.C., Barud H.S., Eceiza A., Tercjak A. (2020). Hydrogels based on waterborne poly(urethane-urea)s by physically cross-linking with sodium alginate and calcium chloride. Carbohydr. Polym..

[19] Chen R.-D., Huang C.-F., Hsu S.-h. (2019). Composites of waterborne polyurethane and cellulose nanofibers for 3D printing and bioapplications. Carbohydr. Polym..

[20] Lee T.J., Kwon S.H., Kim B.K. (2014). Biodegradable sol–gel coatings of waterborne polyurethane/gelatin chemical hybrids. Prog. Org. Coat..

[21] Yang M., Zhang M., Wang Y., Li Y., Han W., Dang X. (2022). Silver Nanoparticle-Loaded Gelatin-Based Nanocomposite Films toward Enhanced Mechanical Properties and Antibacterial Activity. ACS Appl. Bio Mater..

[22] Bele M., Gaberscek M., Dominko R., Drofenik J., Zupan K., Komac P., Kocevar K., Musevic I., Pejovnik S. (2002). Gelatin-pretreated carbon particles for potential use in lithium ion batteries. Carbon.

[23] Wang Y., Zhu T., Kuang H., Sun X., Zhu J., Shi Y., Wang C., Mo X., Lu S., Hong T. (2018). Preparation and evaluation of poly(ester-urethane) urea/gelatin nanofibers based on different crosslinking strategies for potential applications in vascular tissue engineering. RSC Adv..

[24] Kocen R., Gasik M., Gantar A., Novak S. (2017). Viscoelastic behaviour of hydrogel-based composites for tissue engineering under mechanical load. Biomed. Mater..

[25] Pruksawan S., Lim J.W.R., Lee Y.L., Lin Z., Chee H.L., Chong Y.T., Chi H., Wang F. (2023). Enhancing hydrogel toughness by uniform cross-linking using modified polyhedral oligomeric silsesquioxane. Commun. Mater..

[26] Muyonga J.H., Cole C.G.B., Duodu K.G. (2004). Fourier transform infrared (FTIR) spectroscopic study of acid soluble collagen and gelatin from skins and bones of young and adult Nile perch (*Lates niloticus*). Food Chem..

[27] Lian M., Fan J., Shi Z., Zhang S., Li H., Yin J. (2015). Gelatin-assisted fabrication of graphene-based nacre with high strength, toughness, and electrical conductivity. Carbon.

[28] Vieira T., Silva J.C., Borges J.P., Henriques C. (2018). Synthesis, electrospinning and in vitro test of a new biodegradable gelatin-based poly(ester urethane urea) for soft tissue engineering. Eur. Polym. J..

[29] Bankoti K., Rameshbabu A.P., Datta S., Maity P.P., Goswami P., Datta P., Ghosh S.K., Mitra A., Dhara S. (2017). Accelerated healing of full thickness dermal wounds by macroporous waterborne polyurethane-chitosan hydrogel scaffolds. Mater. Sci. Eng. C.

[30] Vashist A., Shahabuddin S., Gupta Y.K., Ahmad S. (2013). Polyol induced interpenetrating networks: Chitosan–methylmethacrylate based biocompatible and pH responsive hydrogels for drug delivery system. J. Mater. Chem. B.

[31] Garrett J.T., Xu R., Cho J., Runt J. (2003). Phase separation of diamine chain-extended poly(urethane) copolymers: FTIR spectroscopy and phase transitions. Polymer.

[32] Shi Y., Zhan X., Luo Z., Zhang Q., Chen F. (2008). Quantitative IR characterization of urea groups in waterborne polyurethanes. J. Polym. Sci. Part A Polym. Chem..

[33] Li S., Kong X., Feng S. (2015). Preparation of uniform poly(urea–siloxane) microspheres through precipitation polymerization. RSC Adv..

[34] Sarkar S., Chourasia A., Maji S., Sadhukhan S., Kumar S., Adhikari B. (2006). Synthesis and characterization of gelatin based polyester urethane scaffold. Bull. Mater. Sci..

[35] Zhang M., Dai Y., Wen L., Wang H., Chu J. (2018). Maskless Surface Modification of Polyurethane Films by an Atmospheric Pressure He/O2 Plasma Microjet for Gelatin Immobilization. Micromachines.

[36] Li Q., Guo L., Qiu T., Xiao W., Du D., Li X. (2016). Synthesis of waterborne polyurethane containing alkoxysilane side groups and the properties of the hybrid coating films. Appl. Surf. Sci..

[37] Jalaja K., Naskar D., Kundu S.C., James N.R. (2015). Fabrication of cationized gelatin nanofibers by electrospinning for tissue regeneration. RSC Adv..

[38] Wang S., Li K., Zhou Q. (2020). High strength and low swelling composite hydrogels from gelatin and delignified wood. Sci. Rep..

[39] Jutrzenka Trzebiatowska P., Santamaria Echart A., Calvo Correas T., Eceiza A., Datta J. (2018). The changes of crosslink density of polyurethanes synthesised with using recycled component. Chemical structure and mechanical properties investigations. Prog. Org. Coat..

[40] Peng L., Wang H., Dai H., Fu Y., Ma L., Zhu H., Yu Y., Li L., Wang Q., Zhang Y. (2021). Preparation and characterization of gelatin films by transglutaminase cross-linking combined with ethanol precipitation or Hofmeister effect. Food Hydrocoll..

[41] Chiellini E., Cinelli P., Grillo Fernandes E., Kenawy el R.S., Lazzeri A. (2001). Gelatin-based blends and composites. Morphological and thermal mechanical characterization. Biomacromolecules.

[42] Wang Z., Liu X., Zhang D., Wang X., Zhao F., Shi P., Pang X. (2015). Coculture with human fetal epidermal keratinocytes promotes proliferation and migration of human fetal and adult dermal fibroblasts. Mol. Med. Rep..

[43] Choi S.-M., Lee M.-W., Shin E.-J. (2019). One-Pot Processing of Regenerated Cellulose Nanoparticles/Waterborne Polyurethane Nanocomposite for Eco-friendly Polyurethane Matrix. Polymers.

